# Filter based phase distortions in extracellular spikes

**DOI:** 10.1371/journal.pone.0174790

**Published:** 2017-03-30

**Authors:** Dorin Yael, Izhar Bar-Gad

**Affiliations:** The Leslie & Susan Goldschmied (Gonda) Multidisciplinary Brain Research Center, Bar-Ilan University, Ramat-Gan, Israel; College de France, FRANCE

## Abstract

Extracellular recordings are the primary tool for extracting neuronal spike trains in-vivo. One of the crucial pre-processing stages of this signal is the high-pass filtration used to isolate neuronal spiking activity. Filters are characterized by changes in the magnitude and phase of different frequencies. While filters are typically chosen for their effect on magnitudes, little attention has been paid to the impact of these filters on the phase of each frequency. In this study we show that in the case of nonlinear phase shifts generated by most online and offline filters, the signal is severely distorted, resulting in an alteration of the spike waveform. This distortion leads to a shape that deviates from the original waveform as a function of its constituent frequencies, and a dramatic reduction in the SNR of the waveform that disrupts spike detectability. Currently, the vast majority of articles utilizing extracellular data are subject to these distortions since most commercial and academic hardware and software utilize nonlinear phase filters. We show that this severe problem can be avoided by recording wide-band signals followed by zero phase filtering, or alternatively corrected by reversed filtering of a narrow-band filtered, and in some cases even segmented signals. Implementation of either zero phase filtering or phase correction of the nonlinear phase filtering reproduces the original spike waveforms and increases the spike detection rates while reducing the number of false negative and positive errors. This process, in turn, helps eliminate subsequent errors in downstream analyses and misinterpretations of the results.

## Introduction

Extracellular recordings of neuronal activity are one of the primary research tools in systems neuroscience [1–5]. The extracellular signal arises from multiple sources which can broadly be differentiated based on their constituent frequencies. High frequency changes in the extracellular signal stem primarily from the spiking activity of one or more neurons in close proximity to the recording electrode. Low frequency changes, also termed the local field potential (LFP), result primarily from sub-threshold and synaptic changes associated with larger neuronal populations [6,7]. Since each of these components dominates a typical range of frequencies, filtration is an efficient process of separating the raw signal into its constituents. Signal filtration is a crucial pre-process in neurophysiology to enable subsequent analyses. For this reason any unaccounted effects attributed to the filter will impact the reliability of downstream processes.

Signal filtration is used for removal of unwanted components based on their spectral composition while preserving others. Filtration properties are defined by their frequency-based magnitude and phase responses. Based on their frequency magnitude response, which describes the amplification of each frequency, filters can be characterized in terms of their pass (amplification ~1) and stop (amplification ~0) bands. The phase response of the filter, which has typically received significantly less attention, describes the phase shifts of the filtered signal as a function of the frequency. Based on the phase response, filters can be classified as zero-phase (ZP), linear phase (LP) or nonlinear phase (NLP) filters [8]. ZP filters do not change the phase of the signal, LP filters change the phase in a linear manner leading to constant time delay of all the frequencies and NLP filters change the phase in a non-linear manner leading to different time delays of different frequencies.

Online and offline filtration processes in neurophysiology generally aim to reduce the magnitude of unwanted frequencies. For example, high pass filters are commonly used preceding the process of spike sorting to reduce the magnitude of the low frequency components in the signal, and low pass filters are used for extracting the LFP signal [9–11]. Filters may be applied online during different stages of the data acquisition or offline during the data pre-processing and analysis. While the exact procedure of filtration varies between the different stages and systems, its focus remains on reducing the amplitude of unwanted frequencies while generally overlooking the phase changes within the preserved frequencies.

The impact of filtration-induced phase changes has been studied in different fields and contexts [8,12]. However, despite the prevalent use of filters in electrophysiology and their major influence on the obtained signal, this issue has generally been neglected. Phase changes induced by filtration have been addressed in a few studies describing the influence of acquisition systems, filters and electrodes on the acquired neurophysiological signal [13], comparisons to wavelet filtration [14], effects of filtration on electroencephalogram (EEG) and magnetoencephalogram (MEG) signals [15,16] and filtration induced analysis artifacts [17]. A related, but not equal, property of filters is their causality, describing the dependence of their output on their input in the time domain. Causal filters use only samples from past and present (t≤0), while non-causal filters may also be dependent on future samples (t>0). In many experimental setups, the requirement for causal filters with minimal time delays results in their implementation using NLP filters and the effect of such causal filters on the phase has been studied [18].

In this paper we focus on the temporal distortion of the spike waveform caused by the usage of NLP filters, which are the de-facto standard used in both online and offline systems in neurophysiology. We demonstrate how these distortions influence downstream analysis and affect both the extracted mean spike shape and spike train. Finally we show how these distortions can be either prevented when the raw (wide-band) signal is available, or corrected in the case of high pass filtered (narrow-band) continuous or segmented data acquisition.

## Materials and methods

### Experimental dataset

**Fig 1:**
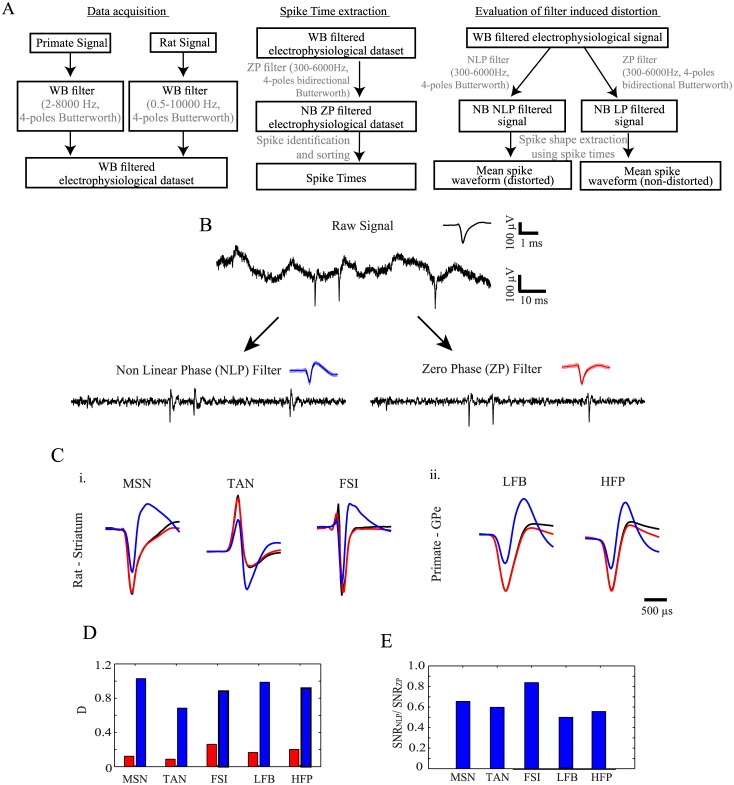
Filtration effect on spike shape in experimental data. (**A**) A scheme presenting the electrophysiological data processing procedures used in this study. (**B)** Raw signal (top) recorded from a rat striatum and the band pass (300–6000 Hz) filtered signals (bottom). The mean action potential waveforms appear as insets (black—raw signal, blue—NLP filtered signal, red—ZP filtered signal. mean ±1 STD). (**C**) Mean waveforms of (**i**) a medium spiny neuron (MSN), a tonically active neuron (TAN) and a fast spiking interneuron (FSI) recorded from the rat striatum. (**ii)** High frequency pauser (HFP) and low frequency burster (LFB) recorded from the primate GPe (**D)** Distance between the raw and post filtration waveforms of the neurons presented in *(B)*. (**E)** The ratio of the SNR of the NLP filtered signal to the SNR of the ZP filtered signal.

The dataset used in this study was collected during studies previously conducted in our laboratory [19,20]. All procedures were in accordance with the National Institutes of Health Guide for the Care and Use of Laboratory Animals, Bar-Ilan University Guidelines for the Use and Care of Laboratory Animals in Research and the recommendations of the Weatherall Report. All procedures were approved and supervised by the Institutional Animal Care and Use Committee (IACUC). These procedures were approved by the National Committee for Experiments on Laboratory Animals at the Ministry of Health. Electrophysiological signals were recorded from the rat striatum [20] and the primate globus pallidus externus (GPe) [19] using standard recording techniques described in detail elsewhere. In both cases the recorded data was filtered online using *a wide band filter* (rat data—0.5–10,000 Hz and primate data 2–8,000 Hz, in both cases 4-pole Butterworth filter). Single unit spike times were offline extracted from this wide-band sampled signal (Offline Sorter V2.8.8, Plexon, Dallas, TX) following an *offline ZP band-pass filtration* of the raw signal (300–6000 Hz, 4-pole bidirectional Butterworth filter). Neurons were classified into different sub-populations based on their firing rate and pattern, and mean spike waveform. Spike detection and timestamp identification via spike sorting were performed once for each signal using the non-distorted signal. Thus, allowing a comparison between the mean waveforms following the different filtration procedures using the exact same spike times and numbers (Fig 1A).

### Simulations

Spike times were modeled as a time series generated from a homogeneous Poisson process with an imposed absolute refractory period of 1 millisecond. The spike trains were convolved with a waveform composed of the first derivative of an intracellularly recorded action potential [21,22] and summed with pink (1/f) noise [7,23,24].

### Filters

The *digital NLP filters* used in this study were designed to be comparable to commonly used hardware and software filters found in acquisition systems and pre-processing software. The NLP filter used was a 4-pole Butterworth bandpass (300–6000 Hz) filter. The application of the same filter using the signal and its reversed form provided the *ZP filter* used throughout the manuscript. An equivalent *LP filter* was used for comparison and defined as 20^th^ order Hamming window based FIR filter with a similar bandwidth.

### Action potential waveform reconstruction

**Fig 2:**
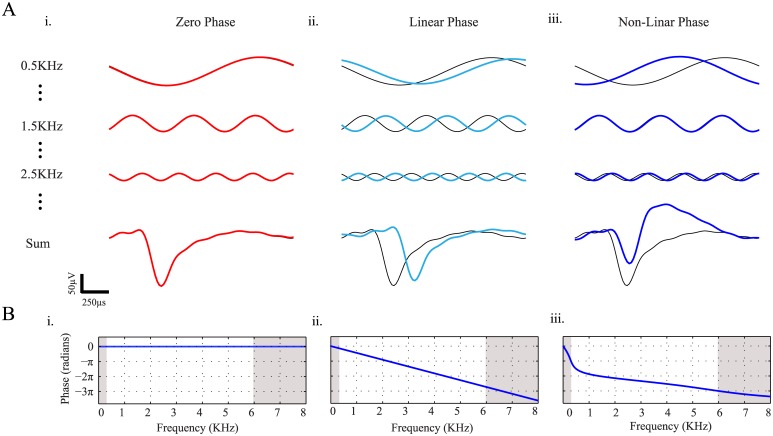
Filter effect on the phase. (**A**) Sinusoidal signals (top) and their summation (bottom) before (black) and after (color) filtration by (i) ZP, (ii) LP, and (iii) NLP band-pass (300-6000Hz) filters. (**B)** Phase responses of the filters used in *(A)*.

The mean action potential waveform represented by 88 samples (2 millisecond window) was transformed using a Discrete Fourier Transform (DFT). The coefficients of the first eight frequencies (0.5–4 KHz) representing the maximal power were used for the reconstruction of sinusoidal functions with amplitudes and phases corresponding to the spike's spectral composition. The spike shape was reconstructed by summing these 8 sinusoidal functions, corresponding to the frequencies, amplitudes and phases of the DFT product (Fig 2).

The MATLAB source code and dataset used in this manuscript is fully available online as supplementary material (http://ibglab.org/software/phaseDistortion.html)

## Results

The extracellular signal is a sum of multiple sources separable by their constituent frequencies (Fig 1B, top). Individual spike times are typically identified by implementing a threshold crossing algorithm that requires pre-processing of the signal in the form of high pass filtering. Most of the online and offline, hardware and software used today in neurophysiology implement analog or digital NLP filters, such as the Butterworth filter, due to their relative efficiency, short time delays and ease of application. However, they impose nonlinear phase shifts that have a major impact on the temporal structure of the filtered signal.

The filtration of an identical extracellular signal using either a NLP filter or an equivalent ZP filter with a similar effect on the amplitudes of different frequencies but no effect on their phases yielded different filtered signals (Fig 1B, bottom). Whereas filtration using a ZP filter maintained the original shape of the action potentials, the spike shape was distorted when the signal was filtered with a NLP filter (Fig 1B). Specifically, the distorted spike shape displayed a lower amplitude trough followed by an additional positive phase which was not present in the original spike shape.

Different neuronal sub-populations display characteristic action potential waveforms that depend on both their cellular properties and the relative location of the recording electrode [22,25]. Neurons recorded from multiple sub-populations in the rat striatum and primate GPe exhibit different distortions when the extracellular signal was filtered using a NLP filter (Fig 1C). The NLP filter led to a severe distortion in all the spike waveforms whereas the magnitude of the distortion and its temporal structure varied across spike shapes. The extent of the waveform distortion was assessed using the Euclidean distance between the waveforms extracted from the raw signal and the waveforms extracted from the filtered signals and normalized by the norm of the mean raw waveform (Fig 1D). The distance between the filtered and the raw waveforms was smaller in the case of a ZP filter than for a NLP filter (0.09–0.26 vs. 0.68–1.03). Additionally, the reduced amplitude of the extremity in the distorted waveform resulted in a reduction of the spike magnitude relative to the background noise. Thus, after NLP filtration, the SNR, defined as the ratio between the maxima of the absolute value of the action potential waveform and the noise standard deviation was smaller for all the neuronal sub-populations than for the SNR after ZP filtration (range 0.49–0.84) (Fig 1E).

The distortion of the spike waveforms can be illustrated by observing the effect of different filters on the temporal delays (phase-shifts) of the pure sinusoidal signals constituting the spike waveform (Fig 2A, top). These delays vary qualitatively between different filters: ZP filters do not change the phase of the different frequencies and thus do not lead to delays in the filtered signal (Fig 2Bi). LP filters have constant time delays for different frequencies due to the linear shift in the phase across frequencies (Fig 2Bii). Finally, NLP filters lead to variable time delays of different frequencies, derived from the nonlinear phase shift as a function of the frequency (Fig 2Biii). Variable time delays across frequencies lead to signal distortion when the signal is composed of more than a single sinusoidal signal with a non-negligible amplitude such as an action potential waveform (Fig 2A, bottom).

**Fig 3:**
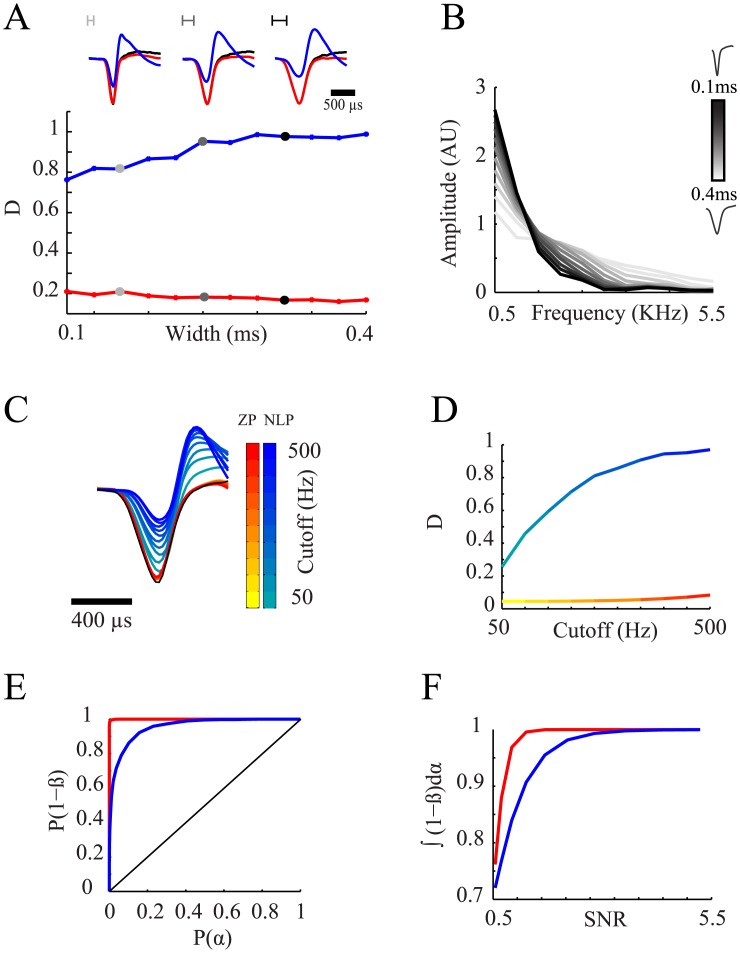
Filtration effect on spike shape in simulated data. (**A**) The effect of the spike width, defined at the half height of the waveform, on the distortion: Top—Examples of waveform distortions for different simulated waveform widths. Bottom—distance between the filtered and raw waveforms as a function of the waveform width. (**B**) Spectral composition of the waveforms referred in (*A*). (**C-D**) The effect of the cutoff frequency on the distortion of the waveform. **(C)** Mean action potential waveforms taken from an identical signal filtered with different filter cutoff frequencies (blue color scale—NLP, red color scale—ZP, black—raw waveform) (**D**) Distance between the filtered and raw waveforms as a function of the filter's cutoff frequency. (**E-F)** Effect of filtration on the detection of action potentials: **(E)** ROC curve of the detection of spikes using different thresholds for NLP and ZP (SNR = 2.7) filtered signals. **(F)** Detection error—the area under the ROC curves as a function of SNR. (red—ZP, blue—NLP).

This non-linearity of the phase response lead to a differential distortion of the spike waveforms depending on their constituent frequencies. We studied the distortion of spikes using simulated signals. The NLP filter induced more pronounced waveform distortions for wider spike waveforms (Fig 3A) since most of their power lies in lower frequencies closer to the filter's cutoff frequency (Fig 3B) which is typically less linear in its phase profile (Fig 2B). To demonstrate the effect of the proximity to the filter's cutoff frequency on the extent of waveform distortion, we filtered an identical signal with a range of cutoff frequencies (Fig 3C and 3D). The waveform distortion following NLP filtration was more pronounced as the cutoff frequency was higher. For all waveforms widths and cutoff frequencies examined, filtration using a ZP filter maintained the original spike shape (Fig 3A–3D).

Waveform distortions thus led to a significant reduction in the waveform amplitude and to a reduction in the SNR, which hindered the process of threshold based spike detection. To examine the effect of filtration induced waveform distortion on the detection of action potentials we compared the rate of false negative (β) and false positive (α) errors in the spike detection for different SNRs in simulated signals compared to the known spike times. For each simulated signal, we used different thresholds for spike detection and calculated the probabilities for α and β errors. These probabilities were then used for the computation of ROC curves, P(1-β) as a function of P(α), for different SNRs. The reduction in SNR following NLP filtration resulted in increased α & β errors (Fig 3E) leading to reduced detection levels for all SNRs (Fig 3F).

**Fig 4:**
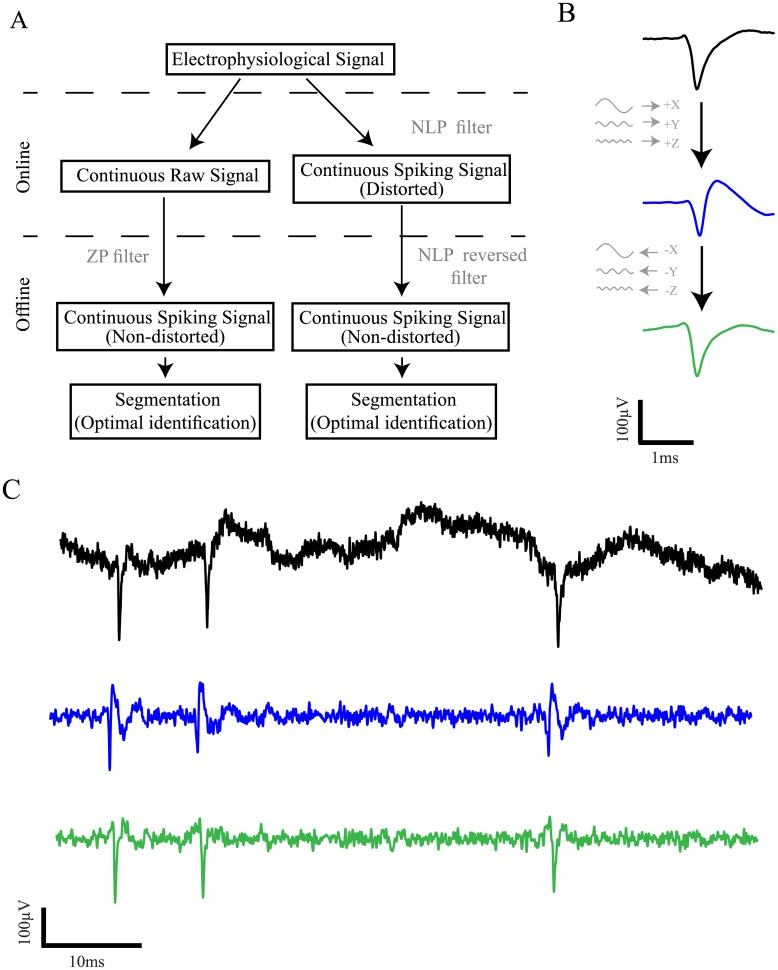
Correction of the waveform distortion in continuous signals. (**A**) A scheme presenting the correction process for different types of data acquisition protocols. (**B)** Illustration of the phase shifts induced by NLP filtration (top arrow) and their shift backwards in the correction process (bottom arrow). The mean waveforms are taken from the signals shown in *(C)*. (**C)** Correction of an extracellularly recorded continuous spiking signal (black—raw signal, blue—filtered signal, green—corrected signal).

Online ZP filtration of the signal during acquisition is not feasible due to its dependency on future inputs. LP filters are typically complex and lead to significant time delays which are intolerable in cases when online processing and feedback are required. Thus, currently, the vast majority of acquisition systems use NLP filters for online filtration. These filters serve to acquire either a wide-band of frequencies, including the classical range of both LFP and spikes (e.g. 1–8,000 Hz, sometimes termed the raw signal), or a narrow-band of frequencies which separates the high frequencies of the signal including primarily the spiking activity (e.g. 300–8,000 Hz, sometimes termed the spiking signal) (Fig 4A). Following the recording of a raw signal, an offline ZP filtration preserving both the time and shape of the signal can be applied to avoid any distortions. However, when the spiking signal is acquired, only the distorted signal is available offline. Theoretically, when the distorted continuous spiking signal is available, shifting each phase backwards to its original location should reverse the waveform distortion (Fig 4B). Based on this principle, given the full description of the NLP filter previously applied to the signal, the phase distortion can be corrected by filtering the reversed acquired signal using the same filter parameters. This process leads to similar phase shifts in the opposite direction, resulting in a zero-phase shift and a restoration of the original waveform (Fig 4C).

**Fig 5:**
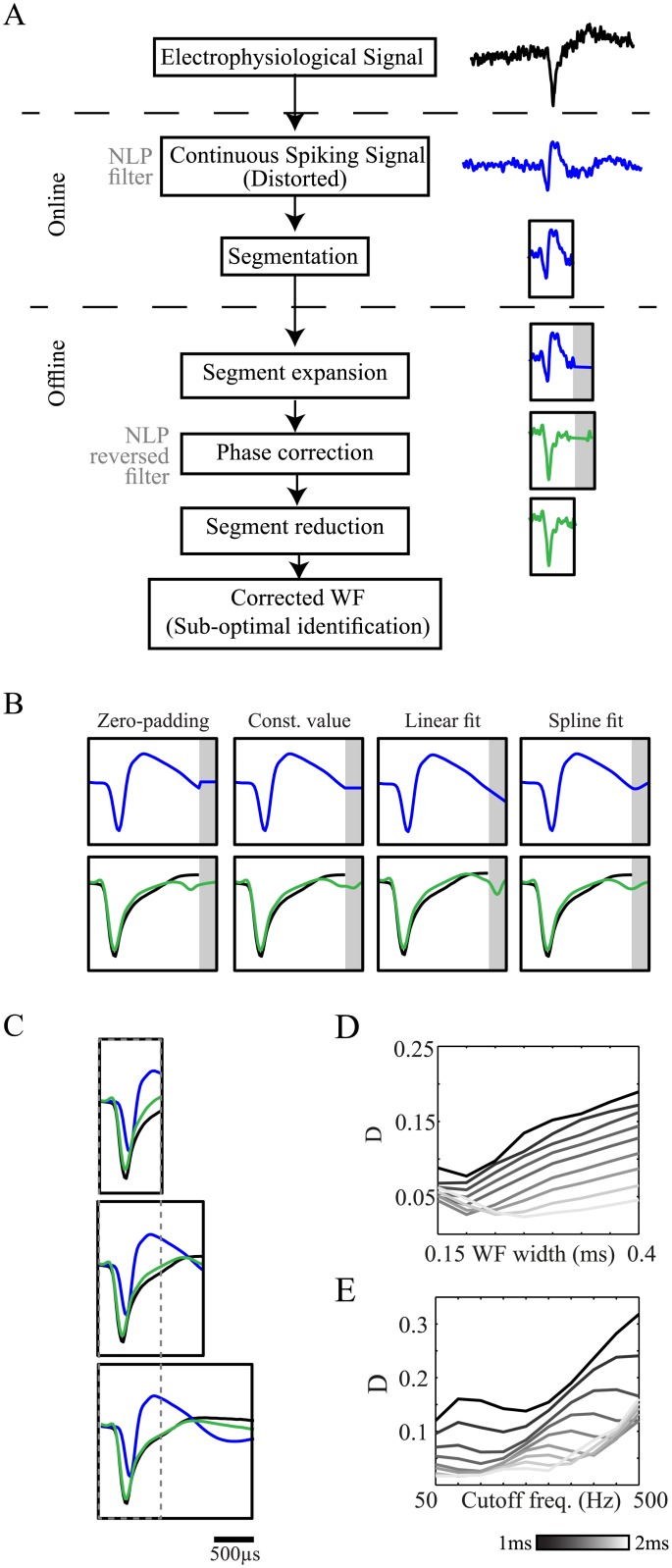
Correction of segmented waveforms distortion. (**A**) A scheme presenting the correction process of segmented waveforms. (**B)** Illustration of different types of segment expansion (top) and the segment correction (bottom) (black—original waveform, blue—NLP filtered waveform, green—corrected waveform, gray area—segment expansion) (**C)** Correction of a segmented signal using linear expansion for different segment widths (black solid frames—window width; gray dashed frames—region of interest in the spike waveform. (**D-E**) The impact of waveform width (D) and filtration cutoff frequency (E) on the correction accuracy for different segment widths (denoted by the scale bar).

In many cases the continuous signal is never acquired but rather only short segments which cross a specific threshold. In these cases of segmented-data acquisition, a retrospect correction of the extracted spike train is not possible. Rather, a partial correction of the waveform by a phase reversal process within the segment is possible (Fig 5A). Reconstruction of the waveform within the short segment is limited by the missing parts of the waveform outside the segment leading to a boundary effect of the waveform ending. A common solution for the boundary effect is padding the edges by zeros (Fig 5B-left, top). However, in many cases, the segmented waveform does not decay to baseline within the segment and thus the zero-padding procedure adds a sharp change which is visible following the reversed filter application (Fig 5B-left, bottom). Different types of expansions to the segment are possible, yielding a better approximation of the missing samples, a reduction of the boundary effect and a more precise correction of the waveform (Fig 5B). The size of the segment directly affects the accuracy of the reconstruction, as wider segments maintain more properties of the waveform. Wider segments enable a more precise representation of the action potential's spectral constituents and a full representation of the spike power, leading to better restoration of the original waveform (Fig 5C). The spectral representation of the segmented waveforms is further dependent on the waveform width and on the cutoff frequency of the filter. In general, the correction accuracy is negatively correlated with the waveform width (Fig 5D), as a similar segment contains larger parts of the waveform. The accuracy is also negatively correlated with the filter's cutoff frequency (Fig 5E) as the phase distortion is largest near this frequency. As a rule of thumb, wider segments yield better waveform correction results negating the effect of segment boundary, waveform width and filtration cutoff frequency. The phase correction of the segmented waveform grossly restores the original waveform of the action potentials and enhances their maximal amplitude and thus the SNR. This may help conduct subsequent analyses; however, it cannot address spikes that were not detected at all due to the reduced SNR prior to segmentation.

## Discussion

In this manuscript we showed that the signal filtration typical of multiple applications in neurophysiology leads to waveform distortions due to nonlinear phase shifts in the filtered signal. This distortion causes a significant change in spike shape and a reduction in the SNR. The reduction of SNR, in turn, leads to errors in spike detection which consequently result in further errors in any analysis that relies on the identification quality. NLP filtration-induced waveform distortion is a significant problem with high impact on both studies interpreting action potential waveforms as well as works analyzing the identified spike trains. Since most acquisition systems and offline analysis software packages use NLP filters, filtration-induced waveform distortion is a widespread problem that should be accounted for in the analysis of extracellularly recorded signals.

Signal distortions caused by nonlinear phase shifts can be either prevented by using LP or ZP filters or retrospectively corrected by deconvolution of the reversed signal using the same filter as when the continuous spiking signal was acquired. However, the acquisition of segmented spiking data is currently the de-facto standard in many applications. Modern multichannel electrode recordings produce large amounts of data requiring excessive storage and processing resources. The amount of stored data may be reduced by an order of magnitude by using online detection and segmentation of the signal. The distortion of the segmented signal as a result of this process is unavoidable and can only be partially remedied retrospectively. Our results show that segmentation and detection based on the distorted signal yields significantly inferior results, primarily due to the reduction in the SNR.

In addition to its use in extraction of spike times from the continuous signal, the action potential waveform is one of the features used for the classification of neurons into different sub-populations [3,20,26,27]. Furthermore, multiple studies use the extracellularly recorded spike shape to extract information about neuronal properties such as the relationship between intracellularly and extracellularly recorded action potentials [22,28], analyze the action potential waveform [29] or model the extracellular spike shape [30–32]. Analysis based on the distorted waveform may lead to a significant misinterpretation of the results and to erroneous conclusions.

NLP filters have been addressed extensively in the general signal analysis literature [8]. In neurophysiology, the issue of filtration-induced signal distortion has received little attention but has been addressed in several studies that observed such distortions. Solutions to these distortions have been suggested in the form of wavelets [14] or usage of linear-phase filters during signal acquisition [33,34]. All of the above solutions are specific cases providing either linear or zero phase response filters and thus allows a prevention of the signal distortion in the time domain. Filter causality, which describes the dependence of its output on its input in the time domain, has been associated with the waveform distortion [18]. However, this relation is not straightforward: while in many experimental setups, the requirement for causal filters with minimal time delays results in their implementation using NLP filters, both causal and non-causal filters can have either linear or non-linear phase effects (see examples of different relations in the supplementary material). Different characteristics of filtration induced distortions have been addressed in studies investigating the impact of the band-pass limitation [35], filtration-induced analysis artifacts [17], the effects of filtration on spiking activity and LFP signals [13] and the effects of filtration and other signal features on spike sorting [36]. Previous studies have also mentioned additional sources of spectral distortions that can arise from the properties of the electrode and acquisition system [13,37] as well as sampling rate and signal resolution [36].

Many researchers in the neurophysiology community are currently oblivious to the impact of the problem created by phase distorting filters on the spiking data that they collect. Thus, while paying close attention to the magnitude modulation of the filters, they are typically unaware of the phase distortions that can have such far reaching consequences. Most of the hardware and software based filtration procedures in both online and offline applications use NLP filters by default, thereby hiding the original waveforms and perpetuating the use of the distorted waveforms. Here we provide a clear description of this problem and emphasize its significance. We present concrete solutions to overcome this problem, which affects the vast majority of neurophysiological studies examining spiking activity through extracellular recordings.
